# Targeting mitochondrial shape: at the heart of cardioprotection

**DOI:** 10.1007/s00395-023-01019-9

**Published:** 2023-11-13

**Authors:** Sauri Hernandez-Resendiz, Aishwarya Prakash, Sze Jie Loo, Martina Semenzato, Kroekkiat Chinda, Gustavo E. Crespo-Avilan, Linh Chi Dam, Shengjie Lu, Luca Scorrano, Derek J. Hausenloy

**Affiliations:** 1 Duke-NUS Medical School, Cardiovascular and Metabolic Disorders Programme, Singapore, Singapore; 2 National Heart Centre Singapore, National Heart Research Institute Singapore, Singapore, Singapore; 3 Veneto Institute of Molecular Medicine, Padova, Italy; 4 Department of Physiology, Faculty of Medical Science, Naresuan University, Phitsanulok, Thailand; 5 Department of Biology, University of Padova, Padova, Italy; 6 National University Singapore, Yong Loo Lin School of Medicine, Singapore, Singapore; 7 University College London, The Hatter Cardiovascular Institute, London, UK

**Keywords:** Cardiovascular diseases, Acute myocardial ischemia–reperfusion injury, Acute myocardial infarction, Heart failure, Mitochondrial morphology, Cardioprotection

## Abstract

There remains an unmet need to identify novel therapeutic strategies capable of protecting the myocardium against the detrimental effects of acute ischemia–reperfusion injury (IRI), to reduce myocardial infarct (MI) size and prevent the onset of heart failure (HF) following acute myocardial infarction (AMI). In this regard, perturbations in mitochondrial morphology with an imbalance in mitochondrial fusion and fission can disrupt mitochondrial metabolism, calcium homeostasis, and reactive oxygen species production, factors which are all known to be critical determinants of cardiomyocyte death following acute myocardial IRI. As such, therapeutic approaches directed at preserving the morphology and functionality of mitochondria may provide an important strategy for cardioprotection. In this article, we provide an overview of the alterations in mitochondrial morphology which occur in response to acute myocardial IRI, and highlight the emerging therapeutic strategies for targeting mitochondrial shape to preserve mitochondrial function which have the future therapeutic potential to improve health outcomes in patients presenting with AMI.

## Introduction

Cardiovascular diseases (CVD) remain the leading causes of death and disability worldwide [216], with acute myocardial infarction (AMI) and heart failure (HF) that often follow being the main contributors to this healthcare burden [250]. Therefore, novel therapies capable of protecting the myocardium from the detrimental effects of acute ischemia–reperfusion injury (IRI) are needed to reduce myocardial infarct (MI) size and preserve cardiac function to prevent the onset of HF following AMI [103].

Morphological and metabolic alterations in mitochondria are known to be associated with the onset and progression of cardiac diseases including AMI and HF [145, 204]. An imbalance in mitochondrial morphology is known to disturb energy production, mitochondrial reactive oxygen species (ROS) generation, and calcium homeostasis, factors which act in concert to contribute to cardiomyocyte death following acute IRI in the setting of AMI [102, 145, 204]. The complex signaling pathways underlying mitochondrial morphology may offer potential therapeutic targets for preventing mitochondrial dysfunction following AMI, so much so that finely tuning the balance between fission and fusion to preserve mitochondrial shape, may lie at the heart of cardioprotection.

In this article, we review how changes in the balance between mitochondrial fission and fusion affect susceptibility to acute myocardial IRI and highlight mitochondrial morphology as a therapeutic target for cardioprotection and for potentially improving health outcomes in patients with AMI. Although the focus of this article is on the role of cardiomyocyte mitochondria in IRI and cardioprotection, it must be appreciated that those studies investigating the role of mitochondria in the heart at the tissue level may not necessarily be restricting their findings to cardiomyocyte mitochondria given the presence of non-cardiomyocyte cells such as immune cells, endothelial cells, and fibroblasts.

## Mitochondrial morphology in the healthy heart

Normal mitochondrial homeostasis and function is determined by a number of different factors including mitochondrial structure, location, morphology, biogenesis, and mitophagy.

### Mitochondrial structure: membrane and lipid composition

Mitochondria are organelles of endosymbiotic origin that harbor two membranes and two aqueous compartments, the outer mitochondrial membrane (OMM) and the inner mitochondrial membrane (IMM), which divide the organelle into an inner boundary membrane and a cristae membrane [114]. Advances in electron microscopy and computer reconstruction algorithms have revealed the cristae to exhibit both tubular and lamellar forms, reflecting their functional specialization in different metabolic microcompartments [114, 199]. The OMM and IMM show significant differences in lipid composition and permeability. The lipid-rich OMM is generally permeable to ions and small uncharged molecules through pore-forming membrane proteins. Among its constituents, the OMM has a voltage-dependent anion channel that provides a route for metabolic substrates (e.g., pyruvate, glutamate, and malate) and nucleotides (e.g., ADP and ATP) to gain access to the intermembranous space (IMS) [147, 205]. There is no membrane potential across the OMM because of its porosity. Furthermore, the OMM provides a dynamic platform for cell signaling and tethers subcellular compartments to form membrane contact sites, including the endoplasmic reticulum (ER), plasma membrane, lysosomes, peroxisomes, endosomes, and lipid droplets [177, 208]. In contrast, the IMM has restricted permeability, with an electrochemical membrane potential (120–180 mV, negative inside) needed to drive oxidative phosphorylation. The IMM contains specific transporters, and translocases that facilitate the passage of substrates into the matrix, where they are metabolized by enzymes, including those of the tricarboxylic acid cycle and fatty acid oxidation, as well as antioxidant enzymes [117]. In addition, the IMM contains cardiolipin (CL), the signature phospholipid of energy-transducing membranes, which has been reported in rat heart mitochondria to become oxidized during ischemia and reduced upon reperfusion [112].

### Mitochondrial distribution in cardiomyocytes

Advancements in live-cell imaging techniques, including 3D reconstruction and electron tomography, have revolutionized our appreciation of the spatial distribution of three distinct subpopulations of mitochondria within cardiomyocytes [186, 194, 207, 221]: interfibrillar mitochondria (IFM), subsarcolemmal mitochondria (SSM), and perinuclear mitochondria (PNM) [221]. Despite their shared cellular environment, these mitochondrial compartments exhibit remarkable heterogeneity in their morphological attributes and biochemical functionalities. This divergence is particularly noticeable in their responses to metabolic and physiological processes.

For instance, these populations demonstrate differences in protein content [70], redox potentials, signifying variations in their oxidative metabolic activity [186]. IFM in adult ventricular cardiomyocytes are typically oval in shape and organized in longitudinal rows alongside the myofibrils and possess a higher rate of substrate oxidation (approximately 1.5 times) than the other two mitochondrial subpopulations [104]. The close proximity of the IFM to the intense energy-requiring demands of the myofilaments results in higher levels of substrate oxidation and increased activity of key oxidative phosphorylation enzymes, including succinate dehydrogenase and citrate synthase. The SSM located directly beneath the sarcolemma may provide the energy supply for the active sarcolemmal transport of electrolytes and metabolites [23, 25]. PNM typically appear more spherical in shape and are distributed around the nucleus in the cardiomyocyte and provide ATP for nuclear transcription. PNM also regulates various nuclear functions, including modifications of promoters to alter transcriptional complex assembly and mRNA expression [166, 207]. Proteomics studies have demonstrated that IFM and SSM possess variations in protein content and synthesis rates [122, 127]. Isotopic tracer methods and peptide analysis by liquid chromatography–mass spectrometry (LC–MS/MS) allow the measurement of mitochondrial protein synthesis in vivo [33, 70, 203]. The heavy water (^2^H_2_O) method with LC–MS/MS analysis has determined that the turnover rates of SSM proteins are faster in mice (average half-life 17 days) [127] than in rats. In contrast, the mitochondrial protein half-life in rats was significantly lower (average half-life 30 days) [122]—the faster turnover of SMM protein in mice correlated with its higher metabolic rate. Interestingly, ischemic damage appears to be progress more rapidly in the SSM subpopulation when compared to IFM.

Additionally, it has been reported that mitochondrial protein synthesis in SSM subpopulations is 15% faster than IFM [122]. Interestingly myocardial protective effects have been shown mainly in SSM subpopulations [139]. Forty-five minutes of ischemia decreased oxidative phosphorylation through cytochrome oxidase in SSM [140]. Most research on cardioprotective therapies that protect the heart against a greater subsequent ischemic insult suggests that the SSM subpopulation is predominantly impacted due to heightened sensitivity to ischemic conditions and Ca2 + overload [57]. This is attributed to the environment associated with the subsarcolemmal and extracellular spaces. Furthermore, connexin 43, associated with cardioprotection, is reported to exist solely in the SSM of cardiomyocytes, further hinting at the role of SSM in protection [25].

Tracking mitochondrial dynamics by photoactivation of mtPA-GFP has revealed marked differences in mitochondrial fusion and fission between the PNM and IFM populations. The PNM population displays significantly heightened fusion and fission activity compared to the IFM population. Intriguingly, sensitivity to mdivi-1, an inhibitor of mitochondrial fission was more pronounced in PNM. This is an intriguing phenomenon, especially considering the similarity in the distribution of fusion–fission proteins between the IFM and PNM. These findings suggest that despite the shared presence of fusion–fission proteins, PNM demonstrates a more dynamic state of fusion and fission [148]. This implies that other regulatory mechanisms or local conditions within the PNM may promote these dynamic activities, shedding light on nuanced differences in the behavior of distinct mitochondrial populations. An improved understanding of these distinct mitochondrial populations may provide a more informed view of cardiac mitochondrial function and regulation and facilitate the development of targeted therapeutic strategies to address mitochondrial dysfunction in cardiac disorders.

### The mitochondrial shaping proteins

**Fig. 1:**
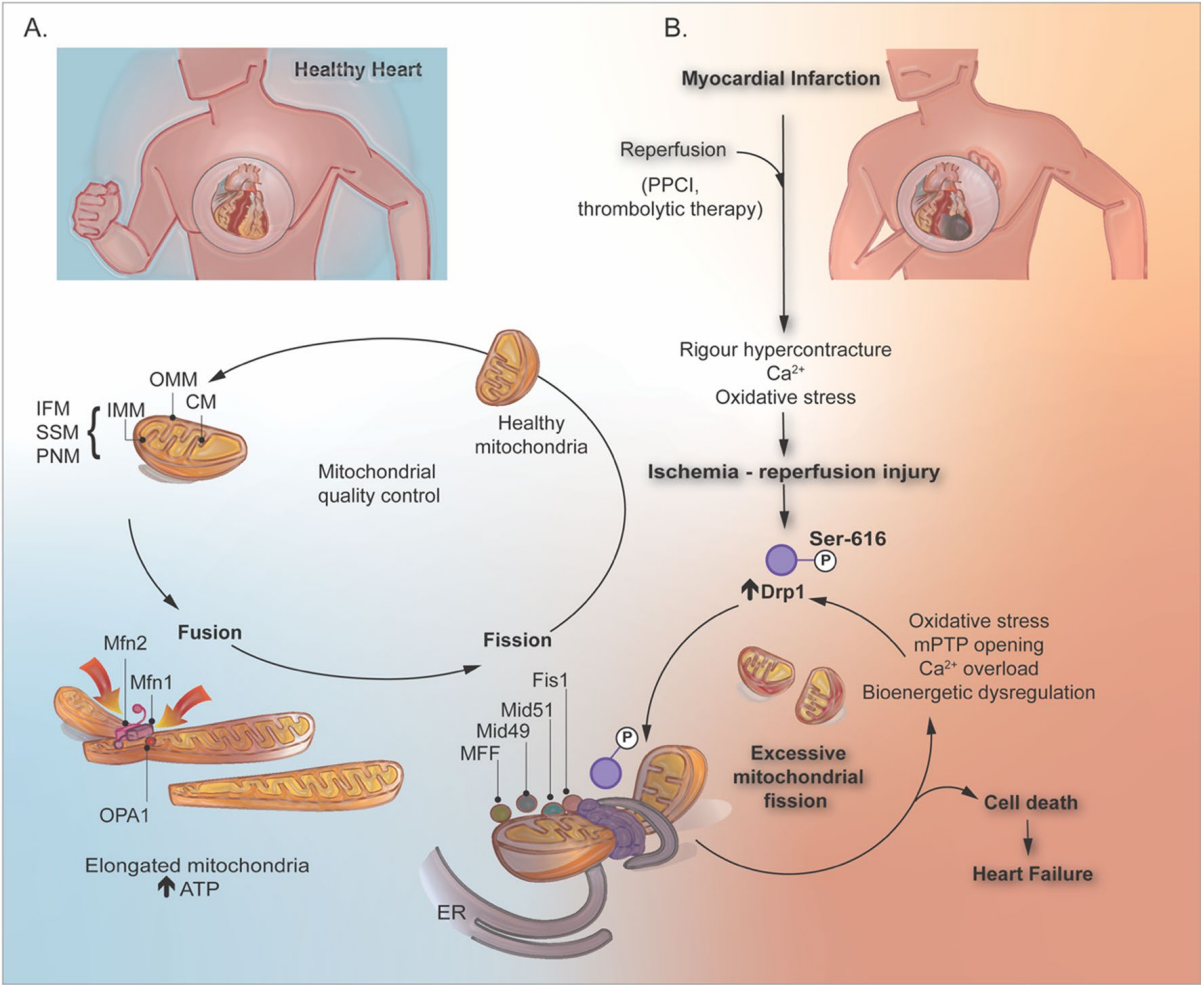
Mitochondrial morphology dynamically adapts to diverse environmental stimuli, resulting in morphological modifications that have implications for cell survival. **A** In healthy hearts, mitochondrial quality control is managed by the dynamic balance between fragmented and elongated phenotypes, enhancing both functionality and metabolism. Mitochondrial fusion, a characteristic process, commences with the interlinking of two proximate mitochondria facilitated by the OMM fusion GTPase proteins, Mfn1 and Mfn2. This action mediates the fusion of the OMM. Subsequently, OPA1 directs the fusion of the IMM and matrix material, culminating in a single elongated mitochondrion. **B** Mitochondrial fission is over-stimulated under IRI conditions, predominantly driven by Drp1 phosphorylation at Ser-616. Adaptor proteins such as Fis1, Mid49, Mid51, and MFF mediate initial mitochondrial constriction preceding Drp1 recruitment. The recruited Drp1 helps form helical ring oligomers, which in turn stimulate the constriction and scission of the outer mitochondrial membrane through GTP hydrolysis. A multitude of upstream signals, some shared and some distinct across cell types, such as cardiomyocytes, endothelial cells, fibroblasts, and vascular smooth muscle cells, incite this excessive mitochondrial fission. This heightened fission often culminates in cellular dysfunction and death, particularly in cardiac tissues subjected to IRI. This figure was created using the Adobe Illustrator 2023. *IRI* ischemia–reperfusion injury, *IFM* interfibrillar mitochondria, *SSM* subsarcolemmal mitochondria, *PNM* perinuclear mitochondria, *OMM* outer mitochondrial membrane, *IMM* inner mitochondrial membrane, *CM* cristae membrane, *Mfn1* mitofusin 1, *Mfn2* mitofusin 2, *OPA1* optic atrophy 1, *Drp1* dynamin-related protein 1, *Mid49* mitochondrial dynamics 49, *Mid51* mitochondrial dynamics 51, *MFF* fission factor, *ER* endoplasmic reticulum, *mPTP* mitochondrial permeability transition pore, *ATP* adenosine triphosphate; PPCI: primary percutaneous coronary intervention

Mitochondria are dynamic organelles that constantly change their shape between a fragmented disconnected phenotype by undergoing fission and an elongated interconnected morphology by undergoing fusion, processes that are coordinated by specific proteins. Mitochondrial fusion plays a vital role in the exchange of genetic material between the mitochondria, enhancing their functionality and resilience, especially under metabolic and environmental stressors (Fig. 1A). Mitochondrial fission is a fundamental process required for dividing organelles and maintaining their quality through mitophagy, ensuring that they function optimally within the cell [98, 178, 180]. In the following section, we provide a detailed description of the cellular machinery involved in orchestrating mitochondrial fusion and fission.

#### Mitochondrial fusion

A series of highly conserved GTPase proteins play vital roles in the dynamics of mitochondrial morphology. Among these, mitofusin 1 (Mfn1) and 2 (Mfn2) are transmembrane GTPases that mediate the fusion process of the OMM [43, 46]. Mfn2 plays an additional role as a tether between mitochondria and endoplasmic reticulum through the interaction of two newly discovered Mfn2 variants ERMIN2 and ERMIT2 [167]. Both Mfn1 and Mfn2 facilitate the docking of two juxtaposed mitochondria through the oligomerization of their GTPase domains, a process which requires guanosine triphosphate hydrolysis [227]. Recent insights into the topology of mitofusins suggest the existence of only one transmembrane domain in human Mfns, suggesting an alternative mechanism for oligomerization of Mfn molecules, which is essential for OMM fusion (Fig. 1) [142, 201]. Mattie et al. showed that two cysteine residues located within the HR2 domains (situated in the IMS) could undergo oxidation when exposed to elevated levels of oxidized glutathione. This oxidation leads to the formation of disulphide bonds between Mfn molecules, facilitating oligomerization. This represents a crucial step in the understanding of the mitochondrial fusion process [157]. Researchers have also demonstrated that introducing glutathione (GSH) to previously formed glutathione disulphide (GSSG)-induced Mfn2 oligomers reversed oligomerization. This novel mechanism underscores the crucial role of redox signaling in OMM fusion [225]. Further investigations are warranted to fully elucidate the mechanistic underpinnings of mitochondrial fusion and understand the impact of aberrant redox signaling on this process.

Post-translational modifications, including phosphorylation, ubiquitination, and deacetylation, modulate Mfns activity. For instance, phosphorylation of Mfn1 in the HR1 domain by extracellular signal-regulated kinase (ERK) inhibits mitochondrial fusion, thereby favoring apoptosis. Conversely, the deacetylation of Mfn2 by histone deacetylase 6 activates it, promoting fusion under conditions of glucose deprivation. Moreover, cellular stress induces the phosphorylation of Mfn2 by JNK, which activates E3 ubiquitin ligase. This ligase ubiquitinates Mfn2, prompting its proteasomal degradation [72]. This intricate interplay of regulatory modifications underscores the complexity of the mitochondrial fusion control mechanisms. The degradation of Mfn2 following its ubiquitination triggers mitochondrial fragmentation and increases the risk of apoptotic cell death. Furthermore, Mfn2 can be phosphorylated by PINK1, a modification that paves the way for its ubiquitination by parkin [86, 98]. This sequence of events culminates in mitophagy, demonstrating the vital role of these processes in regulating mitochondrial dynamics and cellular health.

Another important member of this conserved GTPase family, which belongs to the dynamin class, is optic atrophy 1 (OPA1) [6]. This protein resides in the IMM facing the intermembrane space and regulates mitochondrial fusion of the latter [98] and encompasses eight isoforms in human. These isoforms are generated by alternative splicing [174, 243] of three small exons, namely 4, 4b, and 5b, which are located in the N-terminal region of the gene. Each isoform of OPA1 can feature between one and three proteolytic cleavage sites conventionally labeled S1, S2, and S3 [6]. The S1 site is a common feature of all the eight OPA1 isoforms. In contrast, the S2 and S3 sites each appear in only four isoforms, underscoring the variable proteolytic susceptibility of different OPA1 isoforms. Proteolytic cleavage of the S1 is regulated by the metalloprotease, OMA1. Proteolytic cleavage at sites S2 and S3 is constitutive and is mediated by YME1L [229, 243]. However, cleavage at the S1 site in the OPA1 isoforms is condition specific and occurs exclusively under certain stress-related conditions.

Currently, the precise mechanism of IMM fusion is not fully understood. Ban et al. demonstrated that when recombinant L-OPA1 was incubated with liposomes containing reconstituted CL, a heterotypic interaction was occurring and between L-OPA1 and CL culminating in the fusion of IMM, highlighting the essential role of CL in this process. Further investigation demonstrated that CL is essential for membrane fusion, even when L-OPA1 was present on both sides of the membrane [14, 15]. Ban et al. extended their findings to confirm that the GTPase of OPA1 was necessary to maintain the fusion activity. This implies the pivotal roles of CL-OPA1 binding and OPA1 GTP hydrolysis in IMM fusion [13]. To expand this understanding, further research is necessary to elucidate the exact roles of CL in IMM fusion and to uncover the underlying molecular mechanisms involved. In addition to the proteolytic modifications of OPA1 orchestrated by YME1L and OMA1, there is an additional layer of regulation. Sirtuin-3 (Sirt3), a NAD-dependent deacetylase, targets the GTPase effector domain of OPA1 at lysine residues 926 and 931. This molecular modification increases the GTPase activity of OPA1, thereby promoting an environment conducive to mitochondrial fusion [262]. Other than mitochondrial fusion, OPA1 has a central role in controlling cristae shape in the IMM impinging on mitochondrial metabolism by respiratory chain supercomplexes assembly [54, 239] and on apoptosis, blunting cytochrome c release [79]. We recently found that a redox-insensitive mutant of OPA1 dissociates the mitochondrial fusion and the cristae shape activity of OPA1 [217].

#### Mitochondrial fission

Mitochondrial fission is a critical cellular process in which a single mitochondrion segregates into two distinct entities. This mechanism has several essential functions, including appropriate apportioning and inheritance of organelles during cellular division, ensuring an even distribution of mitochondria within the cell, and facilitating mitophagy and release of cytochrome *c* (cyt*c*), a step integral to apoptosis. If fission is inhibited, the balance between fusion and fission results in the accumulation of elongated damaged mitochondria owing to unopposed fusion activity [98, 202, 262]. Conversely, the disruption of fusion mechanisms results in an overabundance of fragmented mitochondria. The precise mechanisms underlying this phenomenon remain uncertain; however, one plausible explanation suggests that this fragmentation may be a compensatory measure to maintain a consistent ATP supply within the cells.

**Fig. 2:**
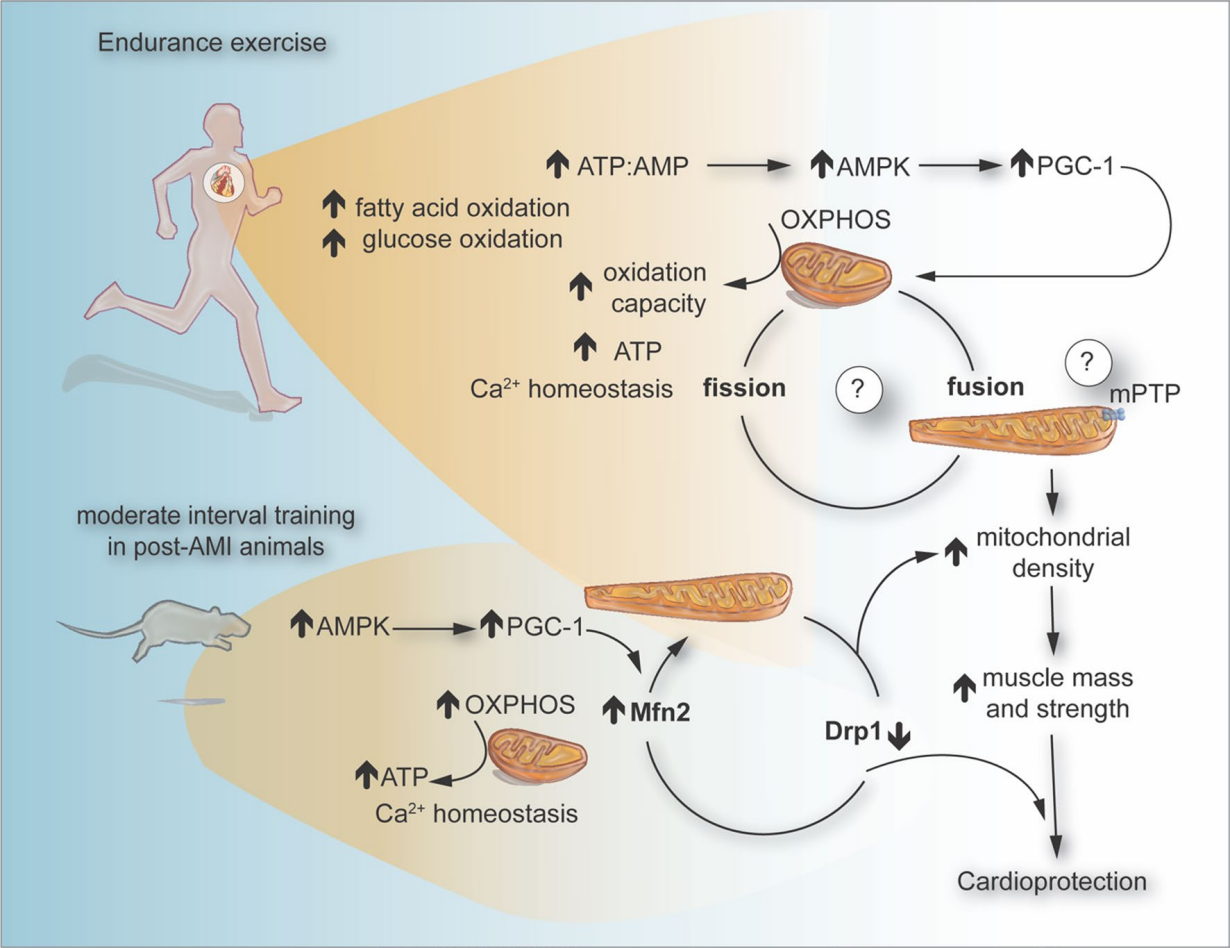
Exercise training is the most accessible and effective intervention for many cardiovascular diseases, primarily because it amplifies the intracellular production of ROS and energy-regulating molecules, such as ATP and AMP. These molecules serve as potent signaling transducers capable of activating a range of protein kinases, including AMPK, an important mediator of glucose and fatty acid oxidation. Moderate interval training has been correlated with cardioprotection against IRI, often attributed to the role of the HSP72, AMPK and PGC-1α. Strikingly, consecutive days of endurance exercise can enhance mitochondrial dynamics in male rats with myocardial infarction by increasing the expression of Mfn2 and PGC-1α, while reducing the levels of Drp1. This figure was created using the Adobe Illustrator 2023. *AMPK* AMP-activated protein kinase, *ROS* reactive oxygen species, *HSP72* heat shock protein 72, *PGC-1α* peroxisome proliferator-activated receptor-*γ* coactivator-1 alpha, *Drp1* dynamin-related protein 1, *Mfn2* mitofusin 2

In mammals, fission is coordinated by dynamin-related protein 1 (Drp1), fission protein 1 (Fis1), mitochondrial fission factor (MFF), and mitochondrial dynamic proteins of 49 kDa and 51 kDa (Mid49 and Mid51) [32]. The preliminary phase of mitochondrial division is facilitated by the ER. In this process, ER tubules establish contact with mitochondria, mediating constriction at these sites prior to the recruitment of Drp1. Once Drp1 is recruited to the outer mitochondrial membrane by the adaptors proteins MFF, Mid49, Mid51, and Fis1, where it forms a ring-like structure around the mitochondrion, amplifying the existing constriction [132, 218]. Subsequently, Drp1 undergoes GTP hydrolysis, leading to the recruitment of dynamin 2 (DNM2) to the site of mitochondrial constriction where it assembles to complete the division process. However, another perspective indicates that DNM2 may not be required for mitochondrial fission and that Drp1 alone, with its constricting and severing capabilities, might suffice to complete the fission process. Whether complete mitochondrial fission occurs in the absence of DNM2 remains to be elucidated [120]. Constriction of the inner IMM is a calcium-dependent process that takes place at the point of contact between the mitochondria and ER. This process is initiated by calcium release from the ER into the mitochondria, leading to IMM constriction and division before the recruitment of Drp1. Notably, CL, in addition to its role in mitochondrial fusion, interacts with Drp1. This interaction promotes the oligomerization of Drp1 and stimulates its GTPase activity, thereby increasing the constriction of liposome membranes. Further research is required to understand how CL modulates the balance between mitochondrial fusion and fission, and the triggers for its diverse roles (Fig. 2) [119].

Drp1 is subjected to an extensive array of post-translational modifications. These include phosphorylation [40], SUMOylation [4], palmitoylation [168], ubiquitination [254], S-nitrosylation [28], and O-GlcNAcylation [51]. These modifications occur predominantly in the B-insert region of the protein and collectively orchestrate the functional regulation of Drp1. Phosphorylation is one of the most extensively studied post-translational modifications of Drp1. Depending on the specific phosphorylation site, it can boost or dampen Drp1 activity. Several sites have been identified, including Ser-579, Ser-40, Ser-585, Ser-44, Ser-592, Ser-656, Ser-616, Ser-637, and Ser-693. Among these, Ser-616 and Ser-637 have garnered scientific attention because of their significant roles in the regulation of Drp1 [200]. Phosphorylation of Ser-616 is an activating event that contributes to the OMM localization of Drp1 and subsequent mitochondrial fission. The most recent kinases known to regulate Drp1 phosphorylation include CDK1/CYCLINB [155], FAK-Erk1/2 [44], AMPK (adenosine monophosphate-activated protein kinase) [69], PINK1 (PTEN-induced putative kinase 1) [224], CAMKII (Ca2 + /calmodulin-dependent kinase II) [245, 255], GSK3ß [141], ROCK1 and DDAH2 (dimethylarginine dimethylaminohydrolase 2)-PKA-NO (nitric oxide) [108].

Focal adhesion kinase (FAK) regulates the phosphorylation of Drp1 via extracellular signal-regulated kinases 1 and 2 (Erk 1/2) in cardiomyocytes. FAK–Erk1/2–Drp1 pathway mediates metabolic adaptation in response to extracellular environment change, and inhibiting this pathway reduces 50% of ATP levels [44]. Chang et al. [44] and Ikeda et al. [111] have demonstrated that FAK–Erk1/2–Drp1 Ser-616 is essential for maintaining the basal energy supply of cardiomyocytes. Fibronectin-activated FAK is associated with mitochondrial fission and respiration via Drp1 Ser1-616 in CMs. However, it has been reported that increased fibronectin expression is associated with cardiac hypertrophy via impaired adrenergic receptors (ARs) [150]. Erk1/2–Drp1 616 activation has been associated with cardiotoxicity in vitro and in vivo rat models. Transient receptor potential cation channel subfamily C member 6 (TRPC6) has been correlated to cardiac pathologies, including MI [149], cardiac hypertrophy [172] and fibrosis [175]. TRPC6–Erk1/2–Drp1 activation induces mitochondrial fission and cell death in a model of (AIC) anthracycline in rat cardiomyocytes [253]. It has been demonstrated that ARs stimulation induces mPTP opening through activating CaMKII via phosphorylation of Drp1 at Ser-616. Inhibiting CAMKII activity or mutating the phosphorylation site Ser-616 rescues cardiomyocytes death by mPTP opening [255].

PINK1 is another kinase that phosphorylates Drp1 at the Ser-616 site, thereby regulating mitochondrial fission [91]. Studies have shown that PINK1 overexpression boosts mitochondrial fission via Drp1 Ser-616, which slows the progression of HFpEF (heart failure with preserved ejection fraction). The same research team found that without PINK1, there is a decrease in genes related to mitochondrial function, membrane potential, and ATP production, pointing to mitochondrial dysfunction. Interestingly, in cells lacking PINK1, the restoration of mitochondrial function was observed with Drp1 overexpression but not with Drp1 Ser-616 [224]. The phosphorylation of the Ser-616 site is vital for Drp1's role in regulating mitochondrial fission and overall function. Consequently, PINK1 acts to phosphorylate Drp1 at this specific site, enhancing mitochondrial performance.

The phosphorylation of Ser-637 prevents the translocation of Drp1 to mitochondria and keeps it inactive in the cytosol thereby preventing mitochondrial fission [261]. The balance of Ser-616 and Ser-637 phosphorylation in Drp1 is not only integral to the function of the protein but also linked to the onset of various diseases [200]. Interestingly, phosphorylation of Ser-616 alone did not induce mitochondrial fission [4] [260]. Given the spatial proximity of Ser-616 and Ser-637 in the three-dimensional structure of Drp1, research has demonstrated that the level of phosphorylation at the Ser-637 site can affect the phosphorylation of Ser-616 [231]. However, the phosphorylation levels at the Ser-637 site were not influenced by the phosphorylation state of Ser-616 [31, 200]. This leads to the intriguing possibility that the basal phosphorylation level of Ser-637 could be instrumental in maintaining the basal phosphorylation state of Ser-616, suggesting a priming role for Ser-637 phosphorylation of Ser-616 [115, 264]. This hypothesis needs to be further tested.

AMPK is an upstream kinase that regulates Drp1 phosphorylation. Intravenous pre-administration with AICAR, an activator of AMPK, improves mitochondrial membrane potential, reduces reactive oxygen species production, and inhibits mitochondrial damage by enhancing phosphorylation of Drp1 at Ser-637 and inhibiting the phosphorylation of Drp1 at Ser-616 [69]. Recently, studies have indicated that DDAH2 modulates Drp1 activity through the nitric oxide synthase (NOS) and subsequent NO generation, leading to Drp1 phosphorylation and mitochondrial fission [108].

### Studying mitochondrial morphology

Our understanding of mitochondrial dynamics has been mainly derived from in vitro studies and non-mammalian models, leaving certain aspects of mammalian mitochondrial function that have not been entirely explored. Factors such as tissue type, specific cell population, and even mitochondrial subpopulations can influence mitochondrial structure and behavior. Yet, the ramifications of this diversity are, in many cases, largely undefined. Consequently, the demand for innovative genetic tools capable of meticulously monitoring dynamic fission–fusion events across diverse tissues, developmental stages, and disease conditions such as cardiovascular diseases is increasingly critical. In this section, we will discuss various strategies for studying mitochondrial dynamics.

Over the last decade, research has recognized the potential of using mitochondrial-targeted fluorescent proteins (FPs) [80, 156, 190], photoactivation [121, 184], and photo-switching [195] to assess mitochondrial dynamics. Genetic tools have also been developed to monitor the dynamic fusion and fission events in different tissues. Several transgenic mouse strains express mitochondria-targeted fluorescent reporters in green- and red-light spectra. These included Mito-EGFP, mtGFP-tg, mtDsRed2-Tg, Acr3–EGFP–CAG–su9-DsRed2, PhAM-excised, Mito-QC, and Mt-Keimareporters. The limitation of green/red spectral emission applies similarly to tissue-specific and inducible reporter strains, including, Mito-Timer, PhAMfloxed, Thy1–mitoDsRed, Thy1–mito-TagRFP, Nse–mitoYFP, CaMKIIa–mitoYFP, Hb9–MitoEGFP, and Endo–mitoEGFP and Thy1.2–mitoDendra [1, 93, 158, 195, 223, 257].

Photoactivation offers external control over the intensity or color of fluorescent emission. This process enables a distinct group of proteins to be marked and tracked, thereby revealing their subsequent dynamics and interactions within individual cells, tissues, and even whole organisms. This precise level of control and visibility presents new opportunities for the in-depth exploration and understanding of biological processes. Two distinct forms of photoactivation have been observed [53, 189, 235]. The first involves reversible photo-switching between the fluorescent and non-fluorescent states, which is brought about by the isomerization of the chromophore. The second is irreversible photoconversion, which occurs due to light-induced covalent modification [7, 184, 195, 248].

Dendra2 (D2) is a monomeric photoconvertible fluorescent protein originally cloned from the soft coral *Dendronephthya sp.*, with a structure similar to that of the green fluorescent protein from the jellyfish *Aequorea Victoria* (avGFP). Similar to avGFP, the unconverted form of D2 showed a peak excitation at 490 nm and a peak emission at 507 nm [84, 185]. However, in D2, short-wavelength light-induced structural photoconversion that shifted the spectral properties to longer wavelengths, with a peak excitation at 553 nm and a peak emission at 573 nm. In contrast to numerous other photo-switchable proteins, the transition from the green (gD2) to the red (rD2) state is irreversible, with the red signal fading solely because of protein degradation. Therefore, D2 fluorescence serves as a robust and enduring marker that enables cells to be tagged and monitored noninvasively across both space and time. The mito-Dendra2 mouse model enables the study of mitochondrial dynamics across a broad range of primary cells and tissues, including disease conditions. Specifically, studies using cardiomyocytes isolated from mito-Dendra2 heart mice demonstrated that mitochondria undergo fission in response to simulated ischemia–reperfusion injury (SIRI). Moreover, it has been observed that hydralazine, a drug commonly prescribed to manage hypertension and heart failure, can prevent mitochondrial fission and reduce MI size. This study underscores the potential of the mito-Dendra2 mouse as a powerful tool for understanding and treating conditions associated with mitochondrial dynamics [195, 197].

Moreover, a diverse range of mitochondrial biosensors have been developed to monitor various processes. These processes include energy production, generation of ROS, redox state, secondary messenger activities (such as those involving cAMP or Ca^2+^), and Zn^2+^ homeostasis. Most of these markers emit fluorescence in the blue and green spectral range. Therefore, the scarcity of ubiquitous mitochondrial reporters that function outside the blue/green/red color spectrum restricts the ability of researchers to track mitochondrial dynamics and other processes visualized using different biosensors [131].

Mito::mKate2, a far-red FPs, has the unique capability to be observed simultaneously with traditional fluorescent markers (including GFP, YFP, CFP, and DsRed), as well as mitochondria-specific biosensors. mito::mKate2 is an effective tool for tracking mitochondrial behavior and cell cycle changes during embryonic development and in adult tissues in mice. The superior brightness and photostability of far-red FPs, such as mito::mKate2, permit a deeper imaging scope than traditional green and red markers. Consequently, mito::mKate2 is better suited for in vivo and ex vivo imaging of mitochondrial activity in living tissues [16].

In parallel with other scientific fields, cutting-edge technologies such as genomics, proteomics, transcriptomics, metabolomics, and epigenomics have spearheaded revolutionary discoveries in mitochondrial biology. The deployment of compartment-specific sensors and techniques for assessing mitochondrial respiration in intact cells has greatly augmented our understanding of mitochondrial physiology. However, despite these knowledge gains, we still face the challenge of comprehensively characterizing and understanding the role of the mitochondria in cardiac diseases. Consequently, it is imperative to expedite the development and application of enabling tools and technologies to bridge the gap between the basic discoveries and their translation into clinical practice.

## Mitochondrial dynamics in cardiac diseases

An imbalance in mitochondrial morphology can impact on energy and mitochondrial ROS production, Ca^2+^ homeostasis, and protein stability, potentially inducing cardiomyocyte death within the heart in a variety of cardiac diseases. In this section, we discuss alterations in mitochondrial morphology linked to cardiac diseases and explore prospective therapeutic strategies aimed at counteracting mitochondrial dysfunction. Such strategies show substantial promise for the prevention and treatment of a range of cardiac conditions.

### Changes in mitochondrial morphology in acute myocardial ischemia–reperfusion injury (IRI)

Currently, the most effective therapeutic intervention for reducing acute myocardial IRI and limiting MI size in AMI patients is timely and effective myocardial reperfusion using either thrombolytic therapy or primary percutaneous coronary intervention (PPCI). However, myocardial reperfusion itself can induce further cardiomyocyte death, a phenomenon known as acute myocardial IRI [101]. The sequence of events that occur during IRI has been extensively explored and has been detailed in several recent reviews [96, 179, 181, 204].

Mitochondria can trigger cell death in cardiomyocytes via two main pathways. The first involves excessive permeability of the OMM, leading to cyt*c* leakage into the cytoplasm. Cytochrome *c* activates caspase-9, which initiates the cleavage of caspase-3. It is characterized by a reduction in mitochondrial membrane potential, increased levels of ROS, increased BAX expression, and decreased Blc-2 expression, a classical route to mitochondria-induced apoptosis [59, 87]. It has been reported that Drp1 acts with Bcl-2 family proteins to accelerate mitochondrial fragmentation and apoptosis. During IRI, Drp1 is recruited to the OMM, instigating the division of these organelles. Several post-translational modifications can affect Drp1 fission activity. In particular, Drp1 phosphorylation at Ser-616 increases its translocation toward the OMM increasing mitochondrial fragmentation and mitochondrial ROS generation. Concurrently, cyt *c* is discharged into the cytoplasm, which triggers an inflammatory response and initiates cell apoptosis [126, 220, 221]. The second pathway is triggered by the sustained opening of the mitochondrial permeability transition pore (mPTP) due to the formation of a non-selective pore in the IMM whose molecular composition is still debated. Prolonged opening of the mPTP induces mitochondrial swelling, collapse of the mitochondrial member potential, and impairment of oxidative phosphorylation, leading ultimately to cell death by necrosis (Fig. 1B) [204].

Mitochondria have been demonstrated to undergo fragmentation during acute myocardial IRI. A study conducted by Ong S. and colleagues revealed that overexpression of Mfn1, Mfn2, or the dominant-negative mutant of Drp1 (Drp1 K38A) to induce mitochondrial elongation delayed the opening of the mPTP and significantly reduce cell death after SIRI in HL-1 cells [182]. Drp1 activation and excessive mitochondrial fission have been observed in peri-infarcted regions of mouse hearts during the initial phase of ischemia [173] and continue to be sustained throughout the reperfusion process [67]. Pharmacological Drp1 inhibition protected adult CMs against simulated IRI, inhibited mPTP opening, and reduced MI size in an in vivo murine model [173].

Mfn2 plays a pivotal role in IRI and HF, given its ability to regulate mitochondrial fusion, ER–ER–mitochondria interaction, cellular metabolism, and cell death. Some studies have suggested that Mfn2 overexpression in heart diseases, such as HF and myocardial ischemia, can mitigate cardiac hypertrophy and dysfunction under various stressors. Conversely, other studies have indicated that deletion of Mfn2 in cardiomyocytes could confer protection against IRI. Thus, there is a pressing need for further research to delve deeper into the detailed molecular mechanisms of Mfn2 in cardiovascular diseases, as it may reveal a potential therapeutic target for patients [50]. Interestingly, acute genetic ablation of both Mfn1 and Mfn2 in murine cardiomyocytes paradoxically reduced MI size following IRI, although one may have expected MI size to be increased due to unopposed mitochondrial fission [89]. The apparent explanation was that the non-fusion pleiotropic effect of Mfn2 as a tethering protein between sarcoplasmic reticulum (SR) and mitochondria had a more dominant effect than the role of Mfn2 on fusion. Therefore, the genetic ablation of Mfn2 protected the mitochondria against mPTP opening and mitochondrial dysfunction by disrupting the association between mitochondria and SR and reducing mitochondrial calcium overload. However, it is worth noting that while acute ablation of Mfn1 and Mfn2 offers protection against acute IRI, long-term ablation of these proteins could be detrimental, leading to cardiomyopathy and sudden cardiac death [89].

Also, OPA1 plays a central role in IRI. A mouse model expressing an increased level of OPA1 displayed protection against cardiac ischemia–reperfusion injury by blunting cristae remodeling and preventing cell death [242]. During ischemia–reperfusion, OPA1 undergoes proteolytic cleavage that is due to the loss of activity of the protein. OPA1 deficiency has been associated with increased sensitivity to IRI with an imbalance in mitochondrial Ca^2+^ uptake [135]. Moreover, increased ROS production occurring during ischemia–reperfusion injury leads to cysteine oxidation of OPA1 contributing to mitochondrial damage and cell death [217].

### Mitochondrial dynamics in heart failure

Heart failure has emerged as a significant health crisis worldwide, particularly in the elderly population. It represents the final stage of a range of cardiovascular diseases and is distinguished by its high incidence rate, frequent hospitalization, and elevated mortality [212, 233]. Heart failure can primarily be categorized into ischemic and non-ischemic types [143]. Ischemic HF is closely linked to coronary artery disease, particularly myocardial infarction, and constitutes approximately 50% of all cases of HF [164].

During the pathological development of HF, cardiomyocytes undergo alterations in their energy metabolism. This transition manifests as an increased dependence on glucose while simultaneously experiencing diminished utilization of fatty acids through beta-oxidation. This metabolic reconfiguration of substrate utilization prompts a shift in cardiac metabolism, reverting it back to a state reminiscent of fetal energy metabolism. When glucose serves as the substrate for energy production, the associated oxygen consumption is reduced compared to when fatty acids are utilized [17]. Severe hypoxia profoundly impairs oxidative phosphorylation. The shift to anaerobic glycolysis fails to produce sufficient ATP to satisfy the energy demands of the heart and leads to lactate accumulation. The depletion of ATP and resultant acidosis contribute to reduced myocardial contractility and damage to membrane pumps and ion channels. These alterations trigger mitochondrial swelling, accumulation of Ca^2+^, and opening of the mPTP. These changes are particularly noticeable in cardiomyocytes affected by myocardial ischemic injury. The role of mPTP has been highlighted as being critical in various forms of cell death associated with myocardial IRI [22, 55, 258]. Indeed, endocardial biopsies from 48 patients, comprising a total of 66 samples, were examined. These patients were diagnosed with cardiomyopathy, a specific form of HF. The analyses revealed diverse cellular architectures, with a notable frequency of changes involving mitochondria. Certain cells exhibit notable mitochondrial changes, including a significant increase in their number, thinning of their matrices, and the occasional emergence of unusually large mitochondria. Initial biochemical investigations were conducted using tissue homogenates from explanted hearts, bypassing the use of isolated mitochondria. These homogenate studies revealed a considerable decrease in both creatine and creatine phosphate levels, implying the depletion of the energy reserves of the cells [10].

Downregulation of Mfn2, a key regulator of mitochondrial dynamics, causes mitochondrial fragmentation, which contributes to the onset of HF. This has been noted both in rat models and patients with pulmonary arterial hypertension (PAH) [215]. In a study conducted by Chen L and colleagues, significant mitochondrial fragmentation was observed in adult Sprague–Dawley (SD) rats with HF post-myocardial infarction. Despite steady levels of OPA1 mRNA, there was a noticeable decline in the protein content of OPA1. In contrast, the protein contents of Mfn1 and Mfn2 remained unaltered [49]. Other studies have shown that in the HF dog model, mitochondrial fission and fusion proteins in the left ventricular myocardium are dysregulated. The expression levels of Drp1 and Fis1 were significantly upregulated. Research has indicated that mice with mutations in the Mff gene experienced mortality at 13 weeks, which is attributable to HF induced by severe dilated cardiomyopathy. The mutant tissues presented a reduction in mitochondrial density and respiratory chain activity while exhibiting an increase in mitochondrial size. These findings suggest that Mff-mediated mitochondrial fission could potentially contribute to the progression of HF [47]. Similarly, a study confirmed that homozygous Mff-deficient (Mffgt) mice exhibited a smaller MI size, restored cardiac function, improved blood flow, and reduced microcirculatory perfusion defects [273].

The inclination toward mitochondrial fission in HF presents several challenges. Primarily, it reduces the number of mitochondria within the cell. This reduction may impair cardiac function owing to diminished ATP production. Second, escalated fission may result in the generation of small, dysfunctional mitochondria. These underperforming mitochondria are not only inefficient in ATP production, but also have a greater likelihood of releasing ROS, which can impact on oxidation of DNA, proteins, and lipids. Along with increased fission, HF induces a decrease in mitochondrial fusion. This decrease in fusion can result in a reduced size and number of mitochondria within the cell, and foster an increase in the aggregation of mitochondria into large clumps. These sizeable mitochondrial aggregations are less efficient in ATP production and more prone to ROS release.

Research into mitochondrial dynamics in HF is currently in its nascent stage, and findings have been mixed, primarily because of two main challenges. First, the complex dynamic system encompassing mitochondrial dynamics and metabolism is involved in the progression of HF, leading to comprehensive studies of the function of mitochondria in HF. Second, mitochondrial dynamics play varying roles in different stages of HF and are influenced by a plethora of pathological conditions. Therefore, more in-depth research is necessary to elucidate the mechanisms underlying mitochondrial dynamics in HF. Such insights could potentially identify critical timing and novel molecular targets, paving the way for the development of innovative therapies for HF. Finally, targeting mitochondrial morphology as a therapeutic strategy for HF will be challenging as it will require chronic treatment which in itself can induce adverse effects such as cardiomyopathy with prolonged inhibition of mitochondrial fission.

### Changes in mitochondrial morphology in other cardiac diseases

#### Diabetic cardiomyopathy (DMCM)

Despite growing evidence highlighting the functional and structural alterations in the myocardium as a consequence of diabetes, the underlying pathological mechanisms, particularly in type 2 diabetic cardiomyopathy (DMCM), remain unclear [249, 256]. DMCM is characterized by abnormal myocardial structure and function in individuals with diabetes, occurring independently of other cardiac risk factors, such as coronary artery disease, hypertension, or significant valvular disease [63, 193, 214]. Recent studies have shown that mitochondrial oxidative damage, mitochondrial dysfunction [76], and diminished cardiomyocyte function [116, 188] are observed in diabetic hearts and contribute to DMCM development. Reduced Mfn2 expression and excessive mitochondrial fission have been demonstrated in diabetic hearts, which results in mitochondrial dysfunction and DMCM. *db/db* mouse hearts showed reduced Mfn2 expression and impaired cardiac function at 12 weeks of age compared with *db/* + mice [76]. Mitochondrial morphological abnormalities, mitochondrial dysfunction, and disrupted Ca^2+^ handling contribute to the development of DMCM [50, 66]. In patients with DMCM, cardiomyocytes exhibit a range of detrimental changes, including fragmented mitochondria and decreased expression of Mfn1. Interestingly, Mfn1 expression was inversely correlated with HbA1c levels, a critical marker of long-term blood glucose control [163, 246]. Bach D et al. showed that mitochondrial fission activity was higher in the hearts of *db/db* mice with type 2 diabetes mellitus, possibly due to diminished Mfn2 expression. The protective effects of Mfn2 in high-glucose and high-fat medium (HG/HF)-treated cardiomyocytes were blunted by fission activation FFCP, while a Mfn2 activator restored mitochondrial fusion and exerted the protective effects in Mfn2-knockdown CMs, suggesting that imbalanced mitochondrial dynamics induced by down-regulated Mfn2 could be the main cause of cardiac dysfunction in diabetic hearts [106]. This was likely linked to a reduction in the expression of peroxisome proliferator-activated receptor α (PPARα) and a subsequent decrease in PPARα binding to the Mfn2 promoter. Given that mitochondrial dynamics serve as the foundation of mitochondrial function, more in-depth investigations are warranted to devise effective interventions targeting mitochondrial fusion and fission in diabetes to retard DMCM progression [12, 106].

#### Hypertension

Hypertension is closely linked to endothelial dysfunction and structural remodeling. Oxidative stress, which is considered a key player in both disease progression and aging, emanates primarily from mitochondria, which are also major targets of ROS [134, 165]. Impairments in mitochondrial biogenesis and dynamics can significantly undermine bioenergetic supply, thereby contributing to endothelial dysfunction and the development of cardiovascular diseases [142]. Activation of the sympathetic nervous system has been recognized as a pivotal factor in the development of hypertension among obese individuals. It also plays a critical role in driving cardiac remodeling processes that occur in association with hypertension. Norepinephrine initiates cardiomyocyte hypertrophy by activating specific signaling cascades, particularly calcium-activated protein phosphatase calcineurin. In hypertensive rats, there was a notable decrease in the mRNA levels of fusion proteins Mfn1, Mfn2, and OPA1 [230]. This suggests a tendency toward increased mitochondrial fragmentation during hypertension. In relation to this, studies on cultured neonatal rat cardiomyocytes treated with norepinephrine have shown it stimulates mitochondrial fission. This event is associated with a decline in mean mitochondrial volume and an increase in the relative number of mitochondria per cell [192]. This change is driven by the norepinephrine-mediated elevation of cytoplasmic Ca^2+^, which in turn activates calcineurin, promoting the relocation of the fission protein Drp1 to the mitochondria. A mutation in Drp1 has been linked to cardiomyopathy, highlighting the essential role of Drp1-mediated processes in preserving normal cardiac function [230, 237]. These findings have led to the speculation that norepinephrine might stimulate mitochondrial fission as a compensatory mechanism to uphold heart contractility under hypertensive conditions, potentially leading to thickening of the ventricular wall. Therefore, it has been proposed that curbing Drp1-mediated mitochondrial fission may help prevent the progression of cardiac pathologies. However, experimental observations also suggest that total loss of Drp1 function may have adverse effects.

Another critical aspect of norepinephrine-induced mitochondrial fission is its connection to both ROS production and cellular apoptosis. It is widely accepted that cyt *c* is released through Bax-lined pores at sites of Drp1-mediated mitochondrial fission, triggering cellular apoptosis [220]. Notably, in the context of hypertension-related left ventricular hypertrophy, both ROS production and myocardial cellular apoptosis are commonly theorized as mechanisms implicated in the onset and progression of the disease. Furthermore, as previously mentioned, hypertension-induced mitochondrial alterations are also associated with changes in mitochondrial energy metabolism, including diminished respiration and ATP production. It has been proposed that, while fusion enhances respiratory efficiency, mitochondrial fission is linked to a decline in oxidative metabolism.

The process of cytosolic Drp1 recruitment to mitochondria during fission is complex and is regulated by post-translational modifications of Drp1. One such regulatory modification is phosphorylation by cyclic AMP-dependent protein kinase A (PKA) at Ser-637 in the GTPase effector domain of Drp1. This action mitigates Drp1 GTPase activity, inhibiting mitochondrial fission. In cardiomyocytes, after a 48-h incubation period with norepinephrine, a reduction in the phosphorylation of Drp1 at Ser-637 was observed. This finding supports the notion that norepinephrine induces mitochondrial fission in cardiomyocytes. Mitochondria predominantly produce ROS, notably superoxide and hydrogen peroxide, which are critical contributors to cellular damage, functional impairment, tissue enlargement, and inflammation in various organs. Hypertension is intricately linked to the reduction and inactivation of the crucial mitochondrial enzyme, sirtuin-3. They play a pivotal role in the management of essential metabolic processes. The absence of sirtuin-3 can precipitate the onset of hypertension and stimulate the progression of cardiac fibrosis, a condition characterized by excess fibrous connective tissue in the heart [64].

Ang II treatment has been observed to significantly enhance the protein expression of Drp1 while inhibiting OPA1 expression in HUVECs. This disruption in mitochondrial dynamics results in cell apoptosis, a process by which acacetin can counteract apoptosis by readjusting the protein expression of Drp1 and OPA1. Other studies have reported similar findings where Ang II provokes the phosphorylation of Drp1 and induces mitochondrial fission in abdominal aortic VSMCs and adventitial fibroblasts, conditions that can be thwarted through Drp1 silencing [62, 107, 209].

#### Obesity

Metabolically unhealthy obesity is linked to an increased risk of obesity-related cardiovascular conditions and overall mortality [19]. The primary cause of obesity is typically identified as an energy imbalance that occurs when calorie intake exceeds the number of calories burned [20, 61, 144]. Evidence suggests that the overconsumption of nutrients can have a detrimental impact on mitochondrial function. Studies have shown that obesity is associated with mitochondrial dysfunction. Introducing chemical uncouplers, such as FCCP or CCCP, triggers complete fragmentation of the mitochondrial network, recruitment of Drp1 to the outer membrane, and degradation of OPA1 [241, 243]. Furthermore, recent research has demonstrated that CCCP-induced depolarization triggers proteasome-dependent degradation of other mitochondrial fusion proteins, including Mfn1 and Mfn2, as well as other outer membrane proteins. Notably, proteasome-dependent degradation of mitofusins necessitates overexpression of the E3–ubiquitin–ligase Parkin [42, 137, 240]. Consistent with this, uncouplers can simulate conditions of excessive nutrient availability, thereby augmenting nutrient oxidation and electron transport chain activity, as observed in activated brown fat or beta cells. Based on this concept, studies involving beta cells subjected to excess nutrients or conditions that decouple the mitochondria under physiological stimuli have demonstrated an upsurge in respiration and pronounced fragmentation within the mitochondrial network [162].

Abundant evidence from both clinical and experimental environments has substantiated the role of obesity in the development of cardiovascular diseases, including HF. Obesity also influences the structure and pumping efficiency of the myocardium, which are notable characteristics of obesity-induced cardiomyopathy [9, 36, 88, 183]. In recent decades, substantial efforts have been made to decipher the intricacies of mitochondrial biogenesis, dynamics, quality control, and their roles in advancing obesity-associated cardiomyocyte dysfunction. Mitochondrial proliferation was increased in *db/db* hearts [30]. A notable morphological shift from a mitochondrial network to fragmented mitochondria has been observed in cardiomyocytes that are affected by obesity. In neonatal rat cardiomyocytes, the initial exposure to palmitate triggers an increase in mitochondrial respiration and heightened mitochondrial polarization and ATP generation. However, prolonged exposure to palmitate (beyond 8 h) produced ROS and induced mitochondrial fission [241]. The occurrence of cardiomyocyte apoptosis and cardiac dysfunction caused by lipid overload may be attributed to changes in post-translational modifications of proteins involved in mitochondrial fission and fusion. This includes an increase in ubiquitination of A-Kinase Anchor Protein 121 (AKAP121), Drp1, and OPA1. The mitochondria and ER are interlinked organelles. Many proteins have been proposed to bind to these two structures at specific locations, known as mitochondria-associated ER membranes. Interestingly, although the disruption of MAMs leads to irregular calcium signaling and cardiac anomalies, a recent study suggested that excessive glucose triggers FUNDC1-mediated mitochondria-associated membrane formation and mitochondrial calcium overload in cardiomyocytes, resulting in functional cardiac abnormalities [251, 252].

Research has confirmed cardiac structure and function alterations in cases of both genetically predisposed and diet-induced obesity [129]. Current understanding of the mechanisms underlying obesity-induced cardiomyopathy includes metabolic disruptions (such as insulin resistance, abnormal glucose transport, increased fatty acids, lipotoxicity, and amino acid imbalance), changes in intracellular calcium homeostasis, oxidative stress, impaired autophagy regulation, myocardial fibrosis, and cardiac autonomic neuropathy (manifesting as either denervation or overflow of adrenergic and renin–angiotensin–aldosterone). Furthermore, factors such as inflammation, small coronary vessel disease (microangiopathy), impaired coronary flow reserve, coronary artery endothelial dysfunction, and epigenetic modifications contribute to the pathogenesis of obesity-induced cardiomyopathy. Although practical targeted medications and procedures are still lacking, a substantial body of research has been devoted to managing obesity-induced cardiomyopathy. Non-pharmacological interventions, such as lifestyle modifications including regular exercise and dietary regulation, could also prove beneficial for cardiac health in individuals with obesity.

#### Aging

Although aging itself is not classified as a disease, it notably affects the functionality of cardiac mitochondria. The research presents differing views, with some studies suggesting a reduction in the number of mitochondria present within the cytoplasm of aged cardiac muscle cells, while others propose that the fraction of cellular volume occupied by mitochondria remains stable throughout the aging process. As we age, the form of the mitochondria changes, becoming less elongated and more spherical. In addition, the surface area of the IMM in the aging heart muscle decreases, although the structure of the cristae, remains unaffected [24, 56, 73, 196]. Generally, older hearts demonstrate less responsiveness to cardioprotective treatments than younger hearts, with all other factors being constant. Aging, a primary risk factor for HF, is linked to the deterioration of nuclear and mitochondrial genetic integrity due to telomere shortening [226]. This process is counteracted by the enzyme telomerase reverse transcriptase. SSM isolated from the heart muscles of aged rodents predominantly preserves their respiration ability. However, IFM exhibited reduced oxygen consumption with age. This decrease in oxygen consumption aligns with the observed decline in the activity of electron transport chain complexes in IFM. Respiratory complex III and IV activities in the IFM of aging heart muscles were diminished. Remarkably, the function of mitochondria remains intact, mainly in aged cardiomyocytes, with their outer membranes disrupted [74, 138]. This age-related decrease in mitochondrial function could influence cellular energy generation, consequently affecting cardiac function. While ATP levels may remain steady in the resting state, evidence from various studies indicates a possible reduction in either ATP content or production [171].

A recently engineered Mito-Timer mouse model revealed a heterogeneous distribution of newly synthesized and aged mitochondria within the heart [234]. Upon examining the expression of proteins integral to mitochondrial fusion and fission, a decrease in the levels of Mfn1 and Mfn2 was observed with age. However, this study found that aging did not affect the OPA1 and Drp1 protein levels [271]. In contrast, a study by Ljubicic et al. showed increased expression of OPA1 and Drp1 with age [146]. These mice demonstrated a build-up of impaired mitochondria, eventually leading to HF. However, moderate catalase expression, explicitly targeted to the mitochondria, normalized ROS production and mitigated structural alterations in hearts deficient in Mfn2. Interestingly, high levels of mitochondrial catalase did not improve mitochondrial function or HF. These data imply that no dose–effect relationship exists between local ROS formation and cardiac degeneration [228].

Progress in mitigating aging-induced health complications will likely hinge on a deeper understanding of the mechanisms that drive aging. In particular, focusing on systems that maintain mitochondrial homeostasis could offer strategies to address mitochondrial damage with aging.

## Targeting mitochondrial morphology in acute myocardial ischemia–reperfusion injury

Currently, therapeutic strategies against acute myocardial IRI that target the mitochondria are mainly focused on the prevention of mitochondrial ROS production and Ca^2+^ overload [98]. In this section, we review cardioprotective interventions aimed at preserving mitochondrial morphology and functionality which may provide new treatment strategies for reduce MI size and preventing HF post-AMI.

### Exercise

Exercise is a nonpharmacological strategy that promotes health and serves as a key strategy for preventing age-related diseases [78, 198]. Notably, exercise induces temporary modifications in the functionality and metabolism of the mitochondria [124]. The influence of exercise training on energy production and its subsequent effects on mitochondrial and metabolic processes have been comprehensively studied. As these adaptations provide insights into the pivotal role of mitochondria in exercise-induced cardioprotection, we will highlight the protective effects of exercise training on cardiac mitochondria in the following section [81, 109].

The metabolic profile of the heart is altered with moderated exercise when compared to the sedentary state. An exercise-trained heart is distinguished by its heightened capacity for fatty acid and glucose oxidation paired with a reduced rate of glycolysis, and the heart boasts a superior capacity to adjust its metabolism in response to acute stress. This adaptability stems from the elevated expression of AMPK, peroxisome proliferator-activated receptor-*γ* coactivator-1 alpha (PGC-1α), and phosphoinositide 3-kinase (PI3K), all of which enhance fatty acid and glucose oxidation, glucose uptake, and the formation of new mitochondria. Engaging in immediate bouts of exercise has been linked to increased production of ROS, primarily as by-products of the electron transport chain [35]. Alleman et al. discovered that energetic mitochondrial recovery, characterized by the oxygen consumption rates following hypoxia–reoxygenation, was enhanced compared to sedentary counterparts in animals subjected to exercise. They also found that the ratio of mitochondrial hydrogen peroxide (H_2_O_2_) production to oxygen consumption was twice as high in mitochondria sourced from sedentary animals than in those from exercised animals. This implies that exercise training may reduce ROS production relative to oxygen consumption. This finding is consistent with other studies, which indicate that exercise training can curb the disruption of the respiratory control ratio in mitochondria exposed to hypoxia–reoxygenation in vitro [5, 8]. Exercise training influences the redox state of cardiac cells and the regulation of Ca^2+^ homeostasis, which could indirectly reduce the susceptibility of mitochondria to IRI. Repeated bouts of endurance exercise protect against IRI arrhythmias, myocardial stunning, and myocardial infarction. Interestingly, only 3–5 consecutive days of endurance exercise is required to achieve a significant level of cardioprotection against IRI [90, 110]. In this line, it has been demonstrated that moderated interval training resulted in improved mitochondrial fusion and fission in male rats with myocardial infarction increasing Mfn2 and PGC-1α and reducing Drp1 (Fig. 2) [11]. However, prolonged exercise resulted in a significant reduction in the gene expression of Mfn1 and Mfn2 and it was an increase in the expression of Fis1 in skeletal muscle of male rats. The magnitude of these alterations was exercise duration dependent. These findings suggest that mitochondrial fusion and fission protein expression are rapidly altered in response to changing energy demand [65]. The direct effect of exercise on mitochondrial dynamics in the heart remains controversial. Future research should aim to discern how exercise training can influence 1) the regulation of mitochondrial dynamics, 2) the control of Ca^2+^ handling and mPTP opening, 3) the intricate interplay between inflammation and mitochondria, and 4) the interaction between mitochondria and redox signaling induced by exercise. This could enhance our understanding of cardioprotective mechanisms and pave the way for the discovery of novel cardioprotective pathways.

### Caloric restriction

Another nonpharmacological strategy that promotes health is the decrease in caloric intake without compromising nutritional needs, also known as caloric restriction (CR) [219]. CR represents the most potent and thoroughly researched dietary intervention across a multitude of non-human species [247]. Furthermore, CR has also been demonstrated to confer broad health benefits in humans, whether dietary restriction is adopted by choice or through unavoidable circumstances. A recent meta-analysis of randomized human trials showed that caloric restriction was associated with a reduction in cardiovascular risk. This was associated with a significant decrease in both blood pressure and heart rate [128]. Mild-to-moderate CR has been found to alleviate cardiac dysfunction in various experimental scenarios including, cardiomyocyte hypertrophy, cardiac fibrosis, inflammation, and mitochondrial damage in middle-aged and aged mice [21, 169, 170].

Short- and long-term caloric restriction also offered protective benefits against acute myocardial IRI [71, 123, 161, 211, 222], and ischemic conditioning mitigates post-ischemic dysfunction in isolated perfused hearts from food-restricted aging rats [2]. However, this effect was not observed in the hearts of aging rats fed ad libitum [2, 3]. Recent studies have indicated that caloric restriction does not alter the susceptibility to mPTP opening in mitochondria isolated from cardiac muscle [219]. In this line, it is important to recognize that the process of isolating mitochondria from tissues can alter their morphology and distribution within cells [133]. This underscores the necessity for more sophisticated tools and standardized experimental models, specifically tailored for studying mitochondria in the context of cardiovascular diseases. Conversely, research has indicated that caloric restriction enhances the expression of Mfn2 in various organs [41]. During nutrient deprivation, protein kinase A (PKA) is activated and phosphorylates Drp1 keeping the latter within the cytoplasm, thereby maintaining mitochondrial fusion [85].

The widespread adoption of caloric restriction appears improbable given the challenge of sustaining long-term CR in contemporary society. Therefore, initiatives are underway to devise pharmacological alternatives that replicate the effects of CR including, metformin [18], resveratrol [45], and rapamycin [34, 125]. These substances, known as caloric restriction mimetics, can confer the advantageous metabolic, hormonal, and physiological effects of CR without necessitating a change in dietary intake.

### Pharmacological modulators—fission

**Fig. 3:**
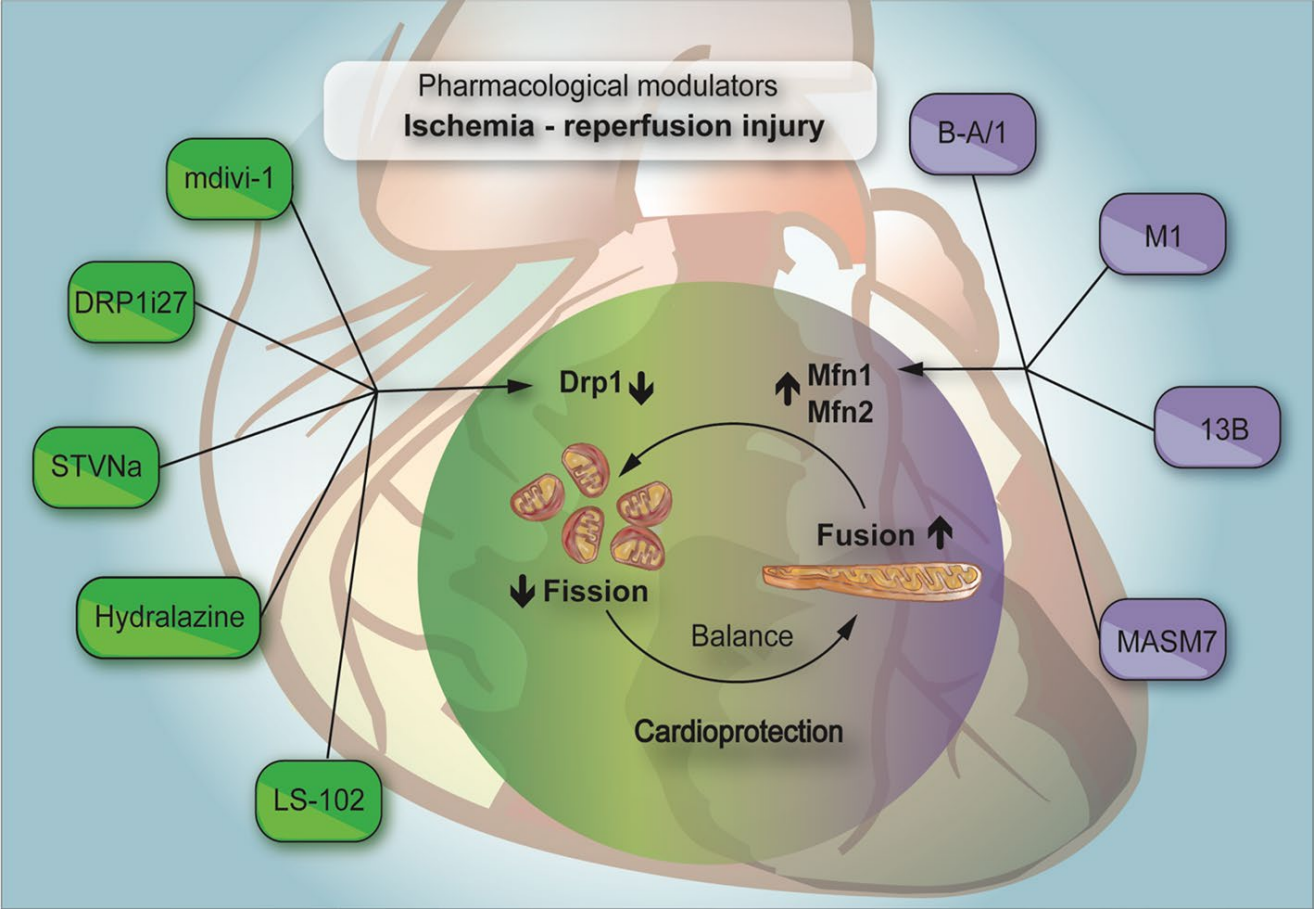
Pharmacological manipulation of mitochondrial fission and fusion processes. Pharmaceutical strategies aimed at modulating mitochondrial dynamics are depicted; those focusing on the regulation of the mitochondrial fission process via strategic manipulation of Drp1 are highlighted in green. In contrast, agents that activate Mfn1 and Mfn2, thereby influencing the fusion process, are shown in purple. This figure was created using the Adobe Illustrator 2023. *Drp1* dynamin-related protein 1, *Mfn2* mitofusin 2, *Mfn1* mitofusin 1

Altered cardiac mitochondrial dynamics with excessive fission are the predominant cause of cardiac dysfunction during IRI. Therefore, several studies have explored the pharmaceutical means for modulating mitochondrial fusion and fission, specifically by manipulating Mfn1, Mnf2, and Drp1 (Table 1, Fig. 3) [98, 154, 180, 181]. Among the available inhibitors, mdivi-1, a quinazoline derivative, is the most extensively studied reversible allosteric inhibitor of Drp1. Mdivi-1 has been demonstrated to effectively inhibit the GTPase activity of Dnm1, a yeast counterpart of Drp1. Its inhibitory activity was observed at a half-maximal inhibitory concentration (IC50) ranging between 1 and 10 μM, indicating its potent inhibitory effect [38]. Ong et al. were one of the first to demonstrate cardioprotection with pharmacological Drp1 inhibition with mdivi-1. Forty minutes of pre-treatment with 50 μmol/L of mdivi-1 decreased mPTP sensitivity and decreased cell death after SIRI in murine cardiomyocytes. A single intravenous bolus of mdivi-1 (1.2 mg/Kg) administered 10 min before acute coronary occlusion significantly reduced myocardial infarct size [182]. In another study by Maneechote et al. investigated the effects of inhibiting mitochondrial fission using mdivi-1. This was performed at three distinct time frames: prior to ischemia, throughout the ischemic phase, and at the initiation of reperfusion, all within the rat cardiac IRI model. The results indicated the most pronounced improvement in cardiac performance when mdivi-1 treatment was implemented before ischemia, which was accompanied by a marked decrease in mitochondrial fragmentation and a notable increase in mitochondrial functionality. Although the administration of mdivi-1 during ischemia and at the onset of reperfusion also resulted in cardiac function enhancement, the level of improvement was comparatively lower than that achieved with the pre-ischemia treatment strategy. Maneechote et al. proposed that the protective effect exerted by mdivi-1 on the left ventricle during IRI incidents might be attributed to its ability to enhance mitochondrial function. They argued that this enhancement was achieved by attenuating excessive mitochondrial fission, which in turn mitigates the incidence of cell death in heart muscle cells or cardiomyocyte death [152]. These preclinical studies indicate the considerable therapeutic potential of Drp1 inhibition. However, the specificity of mdivi-1 has been questioned, highlighting the need for further investigation to validate its selective inhibitory effects [26, 27, 267].

**Table 1:** Pharmacological modulators of mitochondrial fusion and fission

Pharmacological modulator	Mechanism of action	Model	Dose/ concentration range	References
Mdivi-1	Suppresses the GTPase activity of Dnm1, the yeast equivalent of Drp1in mammals	In vitro—yeast In vitro—SIRI HL-1 cells In vivo—IRI in mouse and rat	1–10 µM 50 µM 1.2 mg/Kg (50 µM)	[26, 27, 182, 267]
DRP1i27	Inhibits mitochondrial fission by direct binding to Drp1	In vitro—SIRI in HL-1 cells	5, 10 and 50 µM	[213]
STVNa	Suppresses the activity of mitochondrial fission proteins Drp1 and Fis1	In vitro—SIRI in H9c2 cells	10 µM	[236]
Hydralazine	Diminishes mitochondrial fission by inhibiting the GTPase activity of Drp1	Ex vivo—IRI perfused mouse heart In vivo—IRI mouse heart In vitro— IRI MEFs and CMs	10 mg/kg 1 µM	[118]
LS-102	Reduces mitochondrial fission by enhancing Drp1 Ser-637 and inhibiting GSK3β/Drp1Ser-616	In vivo—IRI in rat In vitro— H9c2 cells	IG 2.5, 5 and 10 mg/kg oral 10 mg/kg50 µM	[48]
AICAR	Suppresses phosphorylation of Drp1 at Ser-616 while promoting the phosphorylation of Drp1 at Ser-637. Inhibits the expression of *Mff* and *Fis1*	Ex vivo—IRI in isolated mouse hearts In vivo—SIRI in H9c2 cells	1 mM	[69]
M1	Promotes mitochondrial elongation by enhancing the expression of Mfn1, Mfn2 and OPA1	In vitro*—*MEFs In vivo—IRI in mouse and pre diabetic rats	5 µM 2 mg/kg	[151, 153, 244]
B-A/1	Induces mitochondrial fusion in MFN2-deficient cells	In vitro— Mfn1 or Mfn2 -null MEFs	1–10 µM	[210]
13 B	Promotes the open and active conformation of Mfn1 and Mfn2	In vivo— Mfn2 KO mice In vivo—CMT2A mice In vitro— MEFs cells	IV 30 ng/g (100 nM) 50 mg/Kg mini osmotic pump 60 mg/kg/day 1 µM, 24 h	[58]
MASM7	Induces mitochondrial fusion through the interaction of HR1–HR2 domains in Mfn1 and Mfn2	In vitro—MEFs cells	1 µM, 2 h	[263]
MFI8	Suppresses mitochondrial fusion by blocking the HR2–HR2 intermolecular interaction and the oligomerization of mitofusins	In vitro—MEFs cells	20 µM, 6 h	[263]

*SIRI* simulated ischemia–reperfusion injury, *MEFs* mouse embryonic fibroblast, *IG* intragastricgavage, *IV* intravenous, *CMs* cardiomyocytes

DRP1i27, a novel small molecule that interacts directly with human isoform 3 of Drp1. Rosdah et al. have demonstrated the protective capabilities of this new molecule. Remarkably, it was shown to shield cells from IRI and toxic conditions, acting in a way which is consistent with the modulatory role of Drp1. The treatment with 50μM of DRP1i27 increased fused mitochondrial networks of mouse fibroblasts in a Drp1-dependent manner. DRP1i27 induced cardioprotection against SIRI in murine atrial HL-1 cells. Additionally, DRP1i27 showed cytoprotective effects against doxorubicin-induced toxicity in human iPSC-derived cardiomyocytes. Insights from molecular docking suggest that DRP1i27 attached to the GTPase site of Drp1, establishing hydrogen bonds with Gln34 and Asp218. The successful identification of DRP1i27 as a binding participant underscores the potential of this compound as a novel small-molecule inhibitor of Drp1 [213].

Another attractive strategy to inhibit fission that has attracted scientific interest is Isosteviol sodium or STVNa [159, 266]. STVNa, a sodium derivative of isosteviol, protects H9c2 cardiomyocytes from IRI by inhibiting the mitochondrial fission pathway [269]. Several studies have examined its diverse therapeutic properties, including the anti-hyperglycaemic, anti-hypertensive, anti-inflammatory, and anti-tumor effects. STVNa effectively maintained mitochondrial membrane potential (Δψ) and notably reduced the overproduction of ROS during reperfusion in a dose-dependent manner. Moreover, compared with diazoxide, a known selective opener of the mitochondrial ATP-sensitive potassium channel reported to safeguard cardiac mitochondria, STVNa presented compelling results [160].

We recently demonstrated the cardioprotective effect of hydralazine [118], a Food and Drug Administration (FDA)-approved therapy for treating essential hypertension, severe hypertension in pregnancy [232], and chronic HF when used in combination with isosorbide-dinitrate [176]. Using photo-switched mitochondrial Dendra2 mice, we demonstrated that pre-treatment with hydralazine inhibited mitochondrial fission, preserved mitochondrial fusion events, and prevented cell death in adult cardiomyocytes following SIRI.

These findings provide new insights into future innovative therapeutic strategies for patients with MI. Future treatments could focus on targeting surplus mitochondrial fission observed during cardiac ischemia or at the initiation of reperfusion, thus providing a potentially effective approach to alleviate the damage caused by such cardiac events.

An imbalance in inositol levels has been reported to affect mitochondrial dynamics and provide valuable insights into the pathogenesis of mitochondrial fission and fusion-related human diseases. Hsu et al. demonstrate that inositol serves as a key metabolite, which directly limits AMPK-dependent mitochondrial fission, independent of its conventional role as a precursor for phosphoinositide creation. A reduction in inositol due to inositol monophosphatase 1 and 2 (IMPA1/2) deficiency triggers AMPK activation and mitochondrial fission, irrespective of ATP levels, whereas inositol accumulation prevents AMPK-dependent mitochondrial fission [105]. Both metabolic stress and mitochondrial damage can lead to decreased inositol levels in cells and mice, thereby inducing AMPK-dependent mitochondrial fission. Inositol directly interacts with AMPK and competes with AMP for this binding, resulting in limited AMPK activation and mitochondrial fission. This research suggests that the AMP/inositol ratio is a pivotal factor in AMPK activation, and proposes a model in which inositol decline is necessary to free AMPK for AMP binding. Therefore, AMPK is an inositol sensor and its deactivation by inositol acts as a mechanism to limit mitochondrial fission. Interventions such as inositol treatment, activation of IMPA1/2, or targeting CDIPT (CDP-diacylglycerol-inositol 3-phosphotidyltransferase)could potentially be effective strategies for addressing a range of human diseases linked to aberrant AMPK-dependent mitochondrial dynamics [105].

The derivative of AS-IV, LS-102, has shown significant efficacy in protecting against IRI damage. LS-102 demonstrated considerable efficacy in reducing apoptosis; reducing the levels of ROS, creatine kinase (CK), lactate dehydrogenase (LDH), and calcium; enhancing the mitochondrial membrane potential; and regulating the Bax/Bcl-2 ratio in cardiomyocytes during IRI. Notably, LS-102 induced IRI-induced mitochondrial fission by reducing the mitochondrial localization of Drp1 via the downregulation of Drp1 phosphorylation at Ser-616 and upregulation of its phosphorylation at Ser-637 in H9c2 cells. LS-102 provides cardioprotection against IRI by inhibiting mitochondrial fission, primarily by blocking GSK-3β-mediated and Drp1 phosphorylation at Ser-616 [48, 206].

### Pharmacological modulators—fusion

Franco et al. first identified mini-peptides derived from Mfn2 that could either specifically activate or inhibit Mfn1 and Mfn2, thereby allowing the manipulation of mitochondrial fusion with the use of mitofusin agonists or antagonists [77]. They proposed that the mechanism of action for the fusion-promoting (agonist) peptide was its ability to compete with intramolecular (between HR1 and HR2 domain) interactions, which normally maintain a closed, non-fusion-permitting conformation. Intramolecular peptide binding results in a more open fusion-friendly conformation. In contrast, the antagonist peptide was thought to function by encouraging the opposite conformational shift, that is, pushing toward a more closed, non-fusion-permitting state. The conformational changes observed in this study were monitored using Mfn2 FRET probes labeled with fluorophores at the amino and carboxyl termini. However, the precise structure of fully intact mitofusin proteins in either a “closed” inactive or “open” active conformations has not been definitively determined and remains a topic of ongoing debate in the field, as discussed earlier [37, 83, 109, 157].

In a subsequent study, Rocha et al. designed a strategy to generate small-molecule peptidomimetics, which demonstrated enhanced in vitro effectiveness over the original mitofusin-activating peptide [210]. They began by identifying a mitofusin-activating peptide with just 11 amino acids. Subsequently, the team used alanine scanning to highlight the amino acids pivotal to the function of the peptide. Finally, they employed a pharmacophore model to facilitate an *in silico* screening process. This study aimed to identify commercially available compounds that shared structural features with amino acids instrumental in the function of the agonist peptide. Biological screening of 55 potential matches led to the identification of two compounds with observable agonist activity. From these, the novel synthesis of what can be termed as "Franken-molecules,” possessing varying chemical segments from these fusogenic compounds, culminated in the creation of the first-of-its-kind small-molecule mitofusin agonist, Chimera B-A/l.

Chimera B-A/l showed the ability to bind to Mfn2 HR2 and replace the original agonist peptide, from which it was designed. Similar to the mitofusin agonist peptide, Chimera B-A/l effectively reversed mitochondrial fragmentation and depolarization in cultured mouse neurons expressing the human Charcot–Marie–Tooth Disease type 2A (CMT2A) mutant Mfn2 protein. Furthermore, this principle mitofusin agonist quickly restored normal axonal mitochondrial trafficking both in vitro, using cultured CMT2A neurons, and ex vivo, using CMT2A mouse sciatic nerves. Collectively, these investigations present an opportunity for future experimental endeavors and potential clinical treatments, leveraging either cell-permeable mini-peptides or small-molecule peptidomimetics that allosterically activate Mfn1 and Mfn2 [210].

M1 is another agent that induces cardioprotection by activating fusion in preclinical models of IRI. M1 has demonstrated significant cardioprotective properties in normal [151] and prediabetic rats [154]. M1 has been shown to restore the expression of mitochondrial fusion proteins, thereby ameliorating mitochondrial function [244]. Another class of small molecule activator of mitofusins with better pharmacokinetics properties has recently been described. This investigation has led to the development of a series of 6-phenylhexanamide derivatives. Through pharmacokinetic optimization, a 4-hydroxy cyclohexyl analog, compound 13, was synthesized. This compound demonstrated potency, selectivity, and oral bioavailability as a preclinical candidate. Intriguingly, further studies of the *cis*- and *trans*- 4-hydroxy cyclohexyl isostereomers of compound 13 revealed that the functional activity and protein interaction were exclusive to the trans-form, referred to as 13 B [58].

Finally, using structural and biochemical insights into the direct modulation of Mfn1 and Mfn2 conformations, Zacharioudakis et al. developed rational pharmacophore methodologies to perform computational screening of small molecules [263]. These strategic screenings yielded small molecules that could either activate or inhibit the fusion activity of mitofusins by modulating their tethering-permissive structure. Their results demonstrated that the mitofusin activator MASM7 and the mitofusin inhibitor MFI8 directly interacted with the recombinant HR2 domain of Mfn2. Moreover, these compounds can also interact with the intact Mfn2 protein found in the mitochondria within cells. MASM7 fosters the pro-tethering structure of Mfn1 and Mfn2, thereby facilitating mitochondrial fusion. In contrast, MFI8 disrupts mitochondrial fusion by actively obstructing the tethering-favorable conformation of mitofusins. The small molecules MASM7 and MFI8 were found to elevate or reduce the levels of GTP-dependent Mfn2 higher-order oligomers. This study establishes a novel strategic avenue for pharmacological intervention with mitofusins using small molecules, thereby enriching the domain of molecular therapeutics. Thus, a deeper exploration of MASM7 and MFI8 in relation to IRI is required [263].

Despite the prevailing challenges, the prospect of applying pharmacological methodologies, either supplementary to or in place of genetic manipulation, opens a new avenue to further understand fusion and fission processes by manipulating their components, which not only enriches the current research, but also potentially holds promise for clinical applications.

### Ischemic conditioning

Ischemic conditioning strategies, including local preconditioning (IPC), postconditioning (IPost), and remote ischemic conditioning (RIC), are potentially promising avenues for therapy, although their mechanisms are not entirely understood and likely involve multiple pathways. IPC, for instance, delays the recovery of intracellular pH, prevents NOS uncoupling, and the subsequent production of reactive oxygen and nitrogen species while amplifying the signaling of protein kinase G (PKG), reperfusion injury salvage kinase (RISK), and survivor activating factor enhancement (SAFE) in reperfused cardiomyocytes. Interestingly, RIC appears to be similar to IPC in that it affects nitrosylation and conserves PKG activity [100] However, RIC also influences mitochondrial function and activates the RISK and SAFE pathways, further expanding its cardioprotective potential [94, 96, 104, 130, 261]

Within the scope of IRI, conditioning strategies aimed at reducing mitochondrial fission or augmenting mitochondrial fusion have been shown to be correlated with diminished IRI. Particularly, remote ischemic conditioning has emerged as a prominent strategy. Heusch [99] accentuates its potential in reducing infarct size among patients with acute myocardial infarction undergoing percutaneous coronary intervention. Meanwhile, Kleinbongard et al. [130] further investigated RIC, examining the various levels of this approach and its associated signal transduction pathways, drawing attention to its successful clinical applications. Chong et al. [52] offered a comprehensive review of the signaling mechanisms associated with RIC, pointing out the divergent outcomes observed in various clinical trials. He further highlighted inconsistent findings in clinical trials, advocating for enhanced research efforts to optimize RIC’s application in cardiac surgery.

The cardioprotective effects of remote RIC are linked to an upregulation in the expression of the mitochondrial fusion protein OPA1 coupled with a reduction in the mitochondrial fission protein Drp1 in the heart. Cellier et al. observed a decrease in Drp1 levels in the mitochondrial fraction following RIC. This implies that RIC can potentially disrupt Drp1 translocation to the mitochondria, thereby obstructing the fission process initiated by ischemia–reperfusion. This suggests that the safeguarding mechanisms of RIC significantly influence the mitochondrial activity dynamics [39]. More recently, we have shown that IPC and IPost preserved the mitochondrial network by inhibiting fission and promoting fusion in H9c2 and adult murine cardiomyocytes subjected to IRI [113].

### Mitochondria-targeted antioxidants

Excessive fragmentation of mitochondria following IRI is a key determinant of mitochondrial damage and cardiomyocyte death [182]. Additionally, mitochondrial dysfunction induced by IRI can result in increased ROS production, which in turn causes more mitochondrial damage and further ROS release, a phenomenon referred to as “ROS-induced ROS release” [97]. Given that mitochondria are the primary source of ROS, scavenging mitochondrial ROS in reperfused cardiomyocytes has long been suggested as a potential therapeutic target for myocardial IRI. Suppressing mitochondrial fission reduces mitochondrial ROS, mitigates mitochondrial dysfunction, and decreases cell apoptosis. Consequently, these effects collectively lead to an improvement in cardiac function.

Melatonin, chemically known as *N*-acetyl-5-methoxytryptamine, is primarily produced by the pineal gland. It plays a multifaceted role in several bodily systems, including immune regulation, the prevention of cancer metastasis, sleep regulation, and circadian rhythms, beyond its basic hormonal functions [92]. Given its antioxidant, anti-inflammatory, and apoptotic properties, melatonin is believed to play a crucial role in mitigating the myocardial damage caused by reperfusion [75, 265]. Preclinical studies have demonstrated that melatonin can inhibit mitochondrial fission under certain pathological conditions [66, 259]. From a mechanistic standpoint, melatonin impedes the mitochondrial translocation of fission proteins, such as the mitochondrial Fis1 and Drp1, as well as the pro-apoptotic protein Bax. Concurrently, it upregulates the expression of mitochondrial fusion proteins, namely Mfn1, Mfn2, and OPA1 [187, 191]. Melatonin inhibits the translocation of Fis1 and Drp1 to the OMM, thereby reducing fission. The mechanisms by which melatonin regulates mitochondrial fusion proteins are intricate and multifaceted. Melatonin may elevate the expression of Mfn1 through Notch1 signaling or, alternatively, it can downregulate both Mfn1 and OPA1 [191, 238]. It has been reported that, melatonin influences the stabilization of Opa1 through the AMPK signaling pathway, and inhibiting AMPK results in decreased OPA1 expression, compromising the cardioprotective benefits of melatonin. In essence, these results validate that OPA1-associated mitochondrial fusion is indeed modulated by melatonin in the context of IRI. Furthermore, orchestrating the AMPK-OPA1-mitochondrial fusion-mitophagy axis via melatonin may represent a novel therapeutic strategy to mitigate myocardial IRI [270].

The clinical intrigue surrounding melatonin as a cardioprotective agent was highlighted in the MARIA trial, which assessed its efficacy in STEMI patients undergoing PPCI [68]. However, it is imperative to approach these results with caution. Notably, the experimental data advocating melatonin’s beneficial impact on MI size primarily originated from small animal MI models devoid of comorbidities and comedications. Much of this research primarily determines melatonin’s long-term effects on post-MI adverse remodeling. More critically, in a pertinent large animal closed-chest reperfused porcine MI model, both intravenous and intracoronary melatonin, when administered pre-reperfusion, did not exhibit a reduction in MI size. This indicates a possible inconsistency in melatonin’s cardioprotective efficacy, even in controlled experimental conditions. Such revelations underscore the need for rigorous vetting of emerging cardioprotective treatments in laboratory settings before transitioning into clinical trials [95].

Resveratrol, a polyphenolic phytoalexin primarily found in grapes, berries, peanuts, and wines, exhibits a wide array of beneficial properties. Chemically identified as 3,4',5-trihydroxystilbene (C14H12O3), it is known for its antioxidant, anti-inflammatory, anti-apoptotic, and anticancer potential. In animal research, resveratrol has been demonstrated to protect cardiomyocytes against oxidative stress, mitigating autophagy, cardiac fibrosis, and apoptosis [268]. Recently, it has been shown that resveratrol promotes the protection of mitochondria from damage caused by hypoxia–reoxygenation events by activating the Sirt1–Sirt3–Mfn2–Parkin–PGC1α pathway [272]. It re-establishes mitochondrial dynamics by accelerating the partitioning of damaged mitochondria and facilitating the interchange of components within the mitochondrial network through the stimulation of fission and fusion processes via the Sirt1–Sirt3 pathway, thereby effectively controlling mitochondrial quantity. Besides, it has been also reported that resveratrol regulates Mfn1 expression via the AMPK pathway and that the inhibition of the MPKA pathway also neutralized the anti-apoptotic effect of resveratrol on re-oxygenated cells [82].

Despite extensive research conducted using animal models, which suggests that antioxidants could potentially be a significant strategy for treating cardiovascular diseases, the therapeutic efficacy of these compounds in humans still requires confirmation. Thus, the field of clinical medicine faces the ongoing task of enhancing our understanding of antioxidants and its viability as a commercially viable therapy, making this an ongoing challenge.

## Summary and conclusions

Changes in mitochondrial morphology in response to acute myocardial IRI are known to mediate mitochondrial dysfunction and cardiomyocyte death providing a therapeutic target for cardioprotection in terms of reducing MI size and preventing HF following AMI. These therapeutic strategies may help restore the balance in mitochondrial fission and fusion perturbed by IRI and could be implemented by acute transient inhibition of mitochondrial fission to preserve mitochondrial function which could potentially be applied at the time of reperfusion following AMI. However, although this treatment approach may be applied to other cardiac diseases characterized by disturbances in the balance of mitochondrial fusion and fission such as diabetic cardiomyopathy and post-AMI HF, the chronic manipulation of mitochondrial morphology such as by sustained inhibition of fission may inadvertently result in cardiomyopathy by allowing the accumulation of damaged mitochondria due to disruption of mitophagy. This may restrict pharmacological manipulation of mitochondrial morphology to acute settings such as for limiting MI size following AMI.

Risk factors, underlying comorbidities, and concurrent medications can significantly impact mitochondrial function, often through multifaceted mechanisms and, in some instances, irreversibly. Consequently, a singular approach targeting only one mechanism for mitoprotection may need to be more holistic [29]. Although the molecular structure of the mPTP remains elusive, and specific inhibitors to deter its activation are yet to be identified, it stands as a promising avenue to counteract reperfusion injury. When it comes to modulating mitochondrial fusion at the onset of reperfusion, a different approach may be needed based on inhibiting Mfn2 to dissociate mitochondria from SR and preventing mitochondrial calcium overload and subsequent mPTP opening and cardiomyocyte death. This supports the notion that targeting singular intracellular components like mPTP or Mfn2 may fall short in cardioprotection. The emphasis should be on a broader understanding of the underlying cardioprotective mechanisms.

Our understanding of how targeting mitochondrial dynamics offers cardioprotection is still evolving. Since the (EU)-CARDIOPROTECTION Cooperation in Science and Technology (COST) discussion in 2019, major advances have been made, and significant molecular information has rapidly accumulated, bringing this classic discipline back to renewed attention. Mitochondrial morphology has been classified under the second category of targets, which includes mechanisms activated endogenously for cardioprotection [60]. Additionally, the introduction of a detailed set of criteria titled 'IMproving Preclinical Assessment of Cardioprotective Therapies (IMPACT) has been stablished to enhance the successful translation of cardioprotective therapies to benefit patients in clinical settings [136].

In conclusion, targeting mitochondrial morphology has the therapeutic potential to reduce MI size and prevent HF following AMI and may also be beneficial in other chronic cardiac conditions characterized by disturbed mitochondrial morphology. However, further studies are needed to investigate the optimal therapeutic approach to manipulating mitochondrial morphology that confers cardioprotection in order that health outcomes following AMI can be improved.
